# Trust in healthcare before and during the COVID-19 pandemic

**DOI:** 10.1186/s12889-023-15716-6

**Published:** 2023-05-11

**Authors:** Helge Skirbekk, Morten Magelssen, Stein Conradsen

**Affiliations:** 1 Department of Nursing and Health Promotion, Faculty of Health Sciences, Oslo Metropolitan University, Oslo, Norway; 2 Department of Undergraduate Studies, Lovisenberg Diaconal University College, Oslo, Norway; 3 Department of Health Management and Health Economics, Faculty of Medicine, University of Oslo, Oslo, Norway; 4 Centre for Medical Ethics, Faculty of Medicine, University of Oslo, Oslo, Norway; 5 MF Norwegian School of Theology, Religion and Society, Oslo, Norway; 6 Faculty of Humanities and Education, Volda University College, Volda, Norway

**Keywords:** Generalised trust, Particular trust, Institutional trust, Street-level bureaucrat, Trust, Healthcare, General practice, Norway, COVID-19, Mandates of trust

## Abstract

**Background:**

Public trust is often advantageous for health authorities during crises such as the COVID-19 pandemic. Norwegian health authorities used the public´s high trust to control the pandemic, resulting in relatively few casualties.

**Methods:**

We wanted to describe and compare the Norwegian public trust in GPs, public healthcare, information and treatment in hospitals before and during the early phases of the COVID-19 pandemic. Further, we wanted to investigate the relationship between somatic or mental illness, and trust in GPs and public health information, and to develop a theoretical understanding of the relationship between trust in healthcare institutions, generalised trust and the societal situation caused by the COVID-19 pandemic. We performed two surveys, the first in December 2019; the second in May 2020, thus providing two snapshots of the Norwegian public’s trust in healthcare and healthcare actors before and during the COVID-19 pandemic.

**Results:**

There was statistically significant increased trust in public healthcare, in treatment at hospital and in information at hospital after the outbreak of the COVID-19 pandemic. There was a non-significant rise in trust in GPs. We found that trust in public health information was not related to mental health nor having a chronic, somatic disease.

**Conclusion:**

The findings confirm that the Norwegian public’s trust in healthcare and healthcare actors is high. The trust levels are also relatively stable, and even show an increase during the early phases of the pandemic. We suggest that there is a dynamic relationship between trust in public health information, healthcare institutions, generalised trust and a societal crisis situation such as the COVID-19 pandemic. However, the GP-patient trust seems less affected by a crisis situation, than the public´s trust in healthcare institutions. This difference may be explained by the relative stability caused by mandates of trust obtained from the patient.

## Background

Public trust can be vital for a government’s ability to handle health crises such as the COVID-19 pandemic. Without trustworthy government communication, the public is less likely to follow official health advice. Several studies find that high levels of public trust in governmental institutions correlate with successful attempts to monitor and control crises [1, 2].

Norway was relatively successful in controlling the COVID-19 pandemic. If we compare numbers of deaths and serious illnesses, Norway was among the least affected nations [3]. We will argue that this success to some extent can be explained by the Norwegian population´s trust in, and willingness to follow, regulations and recommendations from health authorities. In political sciences, it is widely believed to exist a tendency to show support for governments during emergencies (a “rally ‘round the flag” effect). Threats increase a sense of common destiny, and previous studies have found an increased sense of community and trust in the early aftermath of disasters [4]. Further, we will examine the Norwegian population’s trust in healthcare treatment and primary healthcare actors.

In this paper we will investigate the interaction between the public´s trust in general practitioners (GPs), their trust in public healthcare in general, and their trust in information and treatment in hospitals. This investigation will be performed with the early containment of the COVID-19 pandemic in a high trust Nordic country as a background. We will try to accomplish this through a discussion based on results from two survey studies on trust conducted just before and just after the outbreak of the COVID-19 pandemic in Norway.

## Trust during the pandemic in societal context

Although previous research has shown that trust is a fairly stable variable, there are also studies indicating that trust can be both strengthened and weakened during major crises [5, 6]. There is an ongoing debate on the most advantageous levels of trust in a society during a health crises. Some have compared public trust levels and risk perceptions, and others have compared public trust in government institutions with social trust in local communities. While an increased rally ‘round the flag effect is often seen in times of crisis, it is unclear whether this has been the case in the current crisis [6].

In Siegrist’s review on trust and risk perception, it is shown that the relevant issue is not only whether trust is important, but also the form of trust that people rely on in a given situation [7]. Siegrist discusses various trust models and concludes that the importance of trust varies by hazard and respondent group. Another study by Siegrist & al. found that the population’s belief in other people’s trustworthiness, and their belief in information provided by the government, have opposite effects on participants’ risk perceptions [8]. Perceived risks are important for the acceptance of the health authorities implemented measures and for more precautionary behaviour in the public (fewer contacts and better hygiene).

In a study conducted more than 25 years ago (1996) on the relationship between trust and risk perception, Viklund compared Sweden, Spain, United Kingdom, and France [9]. He found that trust was a significant predictor of perceived risk, but the strength of the relationship varied from weak (Spain and France) to moderate (United Kingdom and Sweden). General trust was also a significant source of variation in perceived risk among countries, but much of the variation in perceived risk remained unexplained. Correlations between trust and risk perception also varied depending on the type of risk and trust measure (general trust explained perceived risk better than specific trust). Thus trust may be an element in models explaining risk perception, but its power and the cultural background of the societies studied needs to be considered.

Reiersen & al. claim that during a pandemic, trust can become a double-edged sword [10]. On the one hand, a high level of trust in society may lead to greater acceptance among citizens for public measures that aim to combat a pathogen. Their study compared 127 countries to find that the number of COVID-19 deaths decrease with trust in health authorities (government and science), while the number of deaths increase with social trust (trust in fellow citizens). If the people trust health authorities to implement unbiased and well-informed measures, and expect their fellow citizens to follow these measures, this may lead to a high general compliance - and fewer people might become infected. On the other hand, trust may affect people’s perception of risk, thus influencing their behaviour. The argument is that if people believe that most people are trustworthy, they may be less willing to think of them as a potential health threat. If people also trust the government to manage the pandemic in a competent way, their perception of the risks related to the pandemic weaken. This may lead people in high trust societies to consider personal protective measures and activities less important - and more people will be infected.

Arachchi & Managi’s found that COVID-19 deaths were associated with social capital both positively and negatively [11]. Community attachment and social trust were associated with more COVID-19 deaths, and family bond and security were associated with fewer deaths. COVID-19 deaths were positively associated with population density, ageing population, and interactions between four dimensions of social capital-related factors (community attachment, social trust, family bond, and security) and the ageing population.

A study by Elgar & al. compared 84 countries to show that cross-national differences in COVID-19 mortality relate to income inequality and some dimensions of social capital, even after other cross-national differences (wealth, population size, and population age) were controlled for [12]. Civic engagement and confidence in state institutions related to less mortality, while social trust and group affiliations related to more deaths during the early phase of the pandemic. Risk perception, social trust, and the right balance between health and economic concerns are thus important factors contributing to successful risk management of the COVID-19 pandemic. They conclude that further research is needed on the material and psychosocial pathways that underlie these associations.

Norway is characterised by high levels of trust, both in governing institutions (e.g., police, government and parliament), and generalised trust (expecting fair treatment and trustworthiness from others). The European Social Survey (ESS) and World Values Survey (WVS) both regularly rank Norway and the other Nordic countries on top in surveys of trust [13, 14].

During the COVID-19 pandemic, the high generalised and institutional trust levels in Norway were likely advantageous. Trust was highlighted by the first Norwegian Corona Commission in spring 2021, and several studies previously mentioned show positive relationships between institutional trust, adherence to advice from health authorities, and ultimately reduced mortality [15]. However, some studies show that the effect of trust in the authorities decreases over time, which emphasises the need to follow developments longitudinally [16]. But the causal relationship is not obvious. A strong correlation between high trust and compliance with infection control advice tells us that the most trusting of us are most inclined to comply with advice from health authorities, but we do not know whether a change in trust changes the propensity to comply with advice.

In the early phases of the pandemic, higher trust levels were found among individuals who reported having undergone treatment for COVID-19 [7]. However, the same study also observed that generalised trust in an early pandemic phase did not differ significantly from expected levels based on pre-pandemic measures. This seems to support the hypothesis that trust levels are highly stable and robust against new experiences [17]. Trust in Norwegian governmental institutions increased or remained high during all phases of the pandemic [18]. The high trust levels have been explained as partly due to the Norwegian health authorities’ relatively clear and transparent communication in the face of uncertainty and rapidly changing information during the different phases of the COVID-19 pandemic [19]. Swedish health authorities received lower trust than their Norwegian counterparts during the early phases of the pandemic. This has been explained as a result of lack of transparent communication, and relatively high numbers of deaths and illnesses [20].

Transparent and open public communication have been found to increase trust in many studies [20, 21]. The Norwegian public were exposed to coordinated messages from a limited number of high-ranking government and health officials in mass media almost every day during the pandemic, trying to convey clear and coherent advice. Normally, these were the prime minister, the secretary of health, a top health bureaucrat and a top public health researcher. Differences of opinion were rarely voiced until later, but uncertainties were to a certain degree admitted. The Norwegian population generally accepted and complied with the government´s and the health authorities’ advice and regulations, even though there were protests, and debates in mass media were frequent. Per November 11th 2022, over 91% of the adult population in Norway were vaccinated with at least 2 doses, and almost 94% of the population over 65 have received at least 3 doses. The high vaccination rates and efficient healthcare contributed to the death rates staying relatively low. Out of a population of 5.3 million, there has per November 1st, 2022, been 4286 covid related deaths in Norway, most of them after society opened up in early 2022 (all numbers by FHI-Norwegian Institute of Public Health) [22]. Norway is among the very few countries where life expectancy actually rose during the COVID-19 pandemic [23].

### Trust in healthcare

Patients’ trust in medical doctors has been measured in many studies. In a 2014 study comparing trust in doctors in several countries, trust levels ranged from 83 to 43%. The level of trust in Norway was 72%; the corresponding numbers were 83% in Switzerland, 79% in Denmark, 58% in the USA, and 45% in Russia [24]. Huang & al.’s (2018) study of general trust in doctors in different healthcare systems showed that the degree of commercialisation was negatively associated with trust in medical doctors [25]. This generalised trust in doctors seems to be predicted by income inequality [26, 27].

The patient’s particular trust in doctors, meaning the patients’ individual trust in doctors through direct encounters, is important for the individual healthcare professional’s ability to provide care for patients. Patient trust has an impact not only on patient adherence to advice and treatment, but also on patient satisfaction, and continued enrolment [28–31]. The particular trust in doctors seems, unlike generalised trust, not to be significantly predicted by income inequality [27].

The health conditions of patients play an important role for the degree of particular trust in the healthcare personnel they are in contact with. Plomp & Ballast (2010) performed a mixed-methods study that suggested that patients in a permanent vulnerable situation, such as chronic illnesses, trusted their doctors more than others, but tended to find it difficult to overcome distrust in doctors when the doctor’s independence, agency or expertise was questioned [32]. Reduced mental health is seemingly associated with lower trust in healthcare personnel [33, 34]. For instance, depression is a possible predictor of lower trust in surgeons [35]. In Norway, 16–22% of the adult population meet the criteria for having a mental health condition [36].

Patients’ trust in different healthcare systems is hard to compare. However, there have been many attempts, often using terms such as “confidence in medicine”, or “satisfaction with healthcare system”. In one such study conducted before the COVID-19 pandemic, using the last-mentioned terms, Finland, Great Britain and Denmark scored highest, USA and Sweden lowest, and Norway in the middle [37].

### Trust theories

Trust is a phenomenon that has been investigated within multiple scientific disciplines [38], and has no universally agreed definition. Trust is often defined as the expectation that others will behave with good will, keep promises, and avoid doing harm [39]. Further, it is commonly agreed that trust entails ‘lowering your guard’ and leaving something valuable to others [40]. Information, money and health are examples of such valuables. Mayer, Davis and Schoormans defined trust as the “willingness of a party to be vulnerable to the action of another party based on the expectation that the other will perform a particular action important to the trustor, irrespective of the ability to monitor or control that other party” [41]. In our article, trust should be understood not merely as the outcome of a calculation of risk and assessment [42]. Rather, each instance of trust has its specific dynamic and will unfold differently in different contexts. Hence, trusting a healthcare professional with your health is not necessarily comparable to trusting a bank with your savings, or trusting a friend with secrets.

In the social sciences literature on trust, a differentiation is often made between three types of trust: generalised trust, particular trust and institutional trust [43]. *Institutional trust* refers to institutions such as the healthcare system, the police, parliament, mass media or politicians. *Particular* trust refers to particular individuals (such as family, friends, and your general practitioner). *Generalised* trust refers to expectations towards other people (strangers or people in general). Generalised trust promotes prosocial behaviour and cooperation and correlates with wealthy and peaceful societies [44]. Thus, a high level of trust is normally desirable both for the individual and for society. Nevertheless, optimal trust may not be the same as maximal trust. A very high level of trust can render an individual or a community vulnerable to deception or exploitation, unless combined with realism or scepticism [45].

Some theories try to combine these different types of trust. Michael Lipsky described street-level bureaucrats as professionals that perform their work based on the requirements of the system, yet must also make assessments with discretion being a core part of their everyday business [46]. Thus, particular trust in doctors can be described as a function of the discretionary judgement performed in their encounters with the patients. In interaction with patients, this discretion must balance the interests of both the system, the users and, for that matter, the professionals themselves. A more general expression of institutional contact with citizens is Anthony Giddens’ term of “access points”. An access point is described as the “meeting ground of facework and faceless commitments” [47].

The particular trust aspect of the relationship between healthcare personnel and patients can further be described through the concept “mandates of trust”. This mandate is provided by the patient to the healthcare personnel. The mandate can be “narrow” in a situation where the treatment is specific and time-limited, as in outpatient surgery or acute treatment. A “wider” mandate is necessary if the treatment is more complex, takes place over a greater time span, or entails that the patient shares private information [48].

### Aims

Considering the importance of balanced trust levels in the population, we wanted to study how the Norwegian public’s trust in healthcare and healthcare actors changed before and during the COVID-19 pandemic. Thus, our aims were to:

1. Describe and compare the Norwegian public trust in GPs, public healthcare, information and treatment in hospitals before and after the outbreak of the COVID-19 pandemic.
2. Investigate the relationship between somatic or mental illness, and trust in GPs and public health information.
3. Develop a theoretical perspective of the relationship between trust in healthcare institutions, generalised trust and the societal situation during the very early phases of the COVID-19 pandemic in a high trust society.

## Methods

To investigate our aims, we base our analysis on two surveys. The first was performed in December 2019, two months prior to WHO’s declaration of COVID-19 as a global pandemic; the second in May 2020, at the height of the first wave of the pandemic in Europe. The two questionnaires were part of two different research surveys. The 2019 “Ethical issues in healthcare” survey was initiated by the Centre for Medical Ethics at the University of Oslo. The 2020 survey “Cope COVID-19” was initiated by the Norwegian Centre for Violence and Traumatic Stress Studies (NKVTS). Both surveys included identical items on trust issues. The 2020 survey included these questions with the specific intention of comparing data before and during the pandemic.

Both surveys were performed through web panels by the same data collection agency, Kantar (formerly Norsk Gallup), and drawn from the same panel consisting of approximately 46,000 participants. The Kantar panel was constructed to be representative of the Norwegian general population. The two sets of survey participants were drawn from the panel independently, and so it is not necessarily the same panel members that have been polled twice. Recruitment to the Kantar panel is done by probability sampling, and not self-recruitment. Sampling and weighting are performed based on official statistics from Statistics Norway. Sociodemographic information on panel members is updated each year. The panel is considered representative for the Norwegian ‘internet population’ (everyone who has access to internet), which constitutes about 97% of the total Norwegian population. Panel members are rewarded points for participation according to the number of minutes estimated to complete the questions.

### The context of the studies

At the time of the 2019 study, there were, of course, no pandemic restrictions. At the time of the May 2020 study, the COVID-19 situation was described as under control in the Norwegian society after the initial shutdown March 12th, 2020. The government had started easing the countermeasures. Schools were gradually reopening from May 11th, although most schools did not open for full-day activity until the beginning of June. Most leisure activities were still closed (e.g., gyms, cinemas, museums, theatres), however many institutions aimed to reopen fully or partly during the coming months. The government upheld the rule of physical distance to other people, and advised against non-essential public transport. Employees were instructed to work from home if possible but allowed to attend the office if necessary pending COVID-19 adaptations at the workplace. Our study thus may reflect two snapshots of the Norwegian population: one pre-pandemic, and one intra-pandemic in a period characterised by society ‘opening up’. It should be noted that Norway was not as heavily afflicted by the pandemic as many other countries, as indicated by a relatively low number of fatalities [3]. Additionally, the countermeasures were not among the strictest in a European context; for example, curfews had not been implemented.

### Participants and procedure in the 2019 study

In December 2019, an electronic questionnaire was distributed by Kantar to members of their panel via email. Panel members were given information about the study, which they were told would assess attitudes towards ethical issues in healthcare. 2540 panel members were invited, and 1076 responded. We received 1035 complete responses (response rate 40.7%).

### Measures in the 2019 study

Although most of the 33 items of this questionnaire concerned issues in medical ethics, the questionnaire also contained items on trust in healthcare and healthcare actors. The questionnaire was developed through discussions among the researchers on the project. Lay persons pilot tested the electronic version in two stages.

In both studies, the respondents were asked to rate four items on a scale from 0 to 10, 0 meaning no trust and 10 meaning complete trust. The items were “To what degree do you trust your general practitioner?”, “To what degree do you trust the public healthcare system?”, “To what degree do you trust you will receive correct treatment if you were to become a patient in a hospital?”, and “To what degree do you trust you will receive correct information if you were to become a patient in a hospital?”

### Participants and procedure in the 2020 study

In the 2020 study the response rate was 39.9% (N = 1041).

### Measures in the 2020 study

A number of validated instruments were applied to measure phenomena in terms of mental and somatic health. The questionnaire was briefly piloted before the data collection. The total number of items in the 2020 questionnaire was 95. The four items on trust in healthcare were identical in the 2020 and 2019 study. As in the 2019 study, the respondents were asked to rate the same four items on a scale from 0 to 10, 0 meaning no trust and 10 meaning complete trust.

### Analysis

Statistical analyses were performed with IBM SPSS Statistics version 28. Results are presented with descriptive statistics and mean Likert scores on a five-point scale, with “fully disagree” (= 1) and “fully agree” (= 5) as scale anchors. Univariate and multivariate linear regression was performed for work on research question 2 and 3. Analyses were performed on weighted data to compensate for the sociodemographic skewedness of the data sets. The two samples are in some respect different in terms of socio-demographics. Still, given the size of the samples and that the socio-demographics have been weighted for sociodemographic skewedness, the Independent sample t-test is applicable. Equal variance can be assumed in the two samples for the included variables, except for the “Do you trust that you are given correct treatment if you were admitted to a hospital?” For this variable, we performed the t-test for both assumed equal variances and not assumed equal variances. The figures remained the same in both analysis.

Our discussion follows from abductive reasoning. This entails identifying and piecing together the relevant pieces of evidence, leading towards a coherent picture which is then argued to be the best explanations.

## Results

**Table 1:** Descriptive statistics I

Variable	2019	2020	*Pop (2020)
Age mean	53,5	54,1	39,8
Age groups %			
-49	36,6	51,2	54,0
50–66	37,0	30,3	26,5
67–79	23,5	16,8	14,0
80-	2,9	1,6	5,5
Education %			
Secondary school	4,6	7,1	24,8
Higher secondary school	16,8	19,2	**
Vocational school	13,8	21,8	**
Higher vocational education	9,2	13,6	3,0
University undergraduate	35,7	22,1	24,7
University graduate	24,5	16,2	9,6
Income %			*Pop (2019)
Less than 200.000 NOK	8,3	13,4	18,1
200.000–299.999 NOK	8,6	10,4	14,0
300.000–399.999 NOK	14,4	16,7	15,3
400.000–499.999 NOK	19,8	16,8	14,9
500.000–599.999 NOK	14,8	16,8	**
600.000–699.999 NOK	8,8	8,3	**
700-000–799.999 NOK	6,6	3,9	**
800.000–999.999 NOK	5,8	3,1	**
1.000.000 NOK or more	2,6	1,9	6,3
Chronic disease %		21,0	
Mental health treatment %		24,6	
N (2019) = 1035			
N (2020) = 1041			

*Population figures retrieved from the public records of Statistics Norway

**Figures of these public records are not compatible to those applied in the studies

**Table 2:** Level of trust in healthcare 2019 and 2020. Scale 0–10. Independent sample t-test, two-sided t-test

	2019		2020			
Variable	Mean	S	Mean	S	Mean difference	Sig
Do you trust your GP?	8,04	1,85	8,06	1,96	0,092	0,278
Do you trust the public healthcare service?	7,26	1,74	7,76	1,83	**0,553**	**< 0,001**
Do you trust that you are given correct health information by the Norwegian authorities?^*^			7,86	1,85		
Do you trust that you are given correct treatment if you were admitted to a hospital?	7,53	1,68	7,76	1,81	**0,255**	**0,001**
Do you trust that you will be given correct information if you were admitted to a hospital?	7,31	1,84	7,50	2,04	**0,206**	**0,017**

S = Standard deviation

Sig = Significance at 0.05 level, two-sided p test

Bold = significant result

N (2019 study) = 1035

N (2020 study) = 1041

Weighted for sociodemographic skewedness

*This item was only used in the 2020 study

**Table 3:** Trust in GP by mental treatment, chronic disease, sociodemographic variables. Linear regression. The 2020 study

	Univariate		Multivariate	
Variable	Beta	Sig	Beta	Sig
Mental treatm.*	0,021	0,511	0,051	0,110
Chronic disease**	**0,084**	**0,007**	0,041	0,200
Gender	0,21	0,493	0,029	0,362
Education	-0,010	0,742	0,009	0,771
Age	**0,164**	**< 0,001**	**0,166**	**< 0,001**
Income	0,019	0,549	0,012	0,717

Bold = significant result (P < ,05)

N = 1041 Weighted for sociodemographic skewedness

*The item is: “Have you earlier in your life been treated for mental health issues? Yes/No”.

**The item is: “Do you have a chronic disease or health issue that entails increased risk for severe illness from covid-19 (e.g. cancer, heart issues, diabetes)?”

**Table 4:** Trust in public health information by mental treatment, chronic disease, sociodemographic variables. Linear regression. The 2020 study

	Univariate		Multivariate	
Variable	Beta	Sig	Beta	Sig
Mental treatm.*	-0,054	0,086	-0,028	0,385
Chronic disease**	0,002	0,938	-0,026	0,420
Gender	0,400	0,195	-0,036	0,255
Education	0,007	0,823	0,013	0,694
Age	**0,109**	**< 0,001**	0,**113**	**<0,001**
Income	0,051	0,098	0,018	0,594

Bold = significant result (P < ,05)

N = 1041 Weighted for sociodemographic skewedness

*The item is: “Have you earlier in your life been treated for mental health issues? Yes/No”.

**The item is: “Do you have a chronic disease or health issue that entails increased risk for severe illness from covid-19 (e.g. cancer, heart issues, diabetes)?”

**Table 5:** Trust in healthcare actors by age groups. The 2020 study. Means. Scale 0–10. ANOVA.

		2019			2020		
Variable	Age	Mean	S	Sig.	Mean	S	Sig.
Trust in GP	**-49**	**7,78**	**2,009**	**0,004**	**7,76**	**1,961**	**< 0,001**
	**50–66**	**8,04**	**1,790**		**8,19**	**2,014**	
	**67–79**	**8,34**	**1,760**		**8,63**	**1,704**	
	**80-**	**8,50**	**2,060**		**8,92**	**1,433**	
	**Total**	**7,97**	**1,918**		**8,06**	**1,957**	
Trust in public healthcare	**-49**	**7,12**	**1,839**	**0,001**	**7,54**	**1,919**	**< 0,001**
	**50–66**	**7,09**	**1,755**		**7,86**	**1,764**	
	**67–79**	**7,67**	**1,561**		**8,20**	**1,605**	
	**80-**	**7,64**	**1,532**		**8,43**	**1,418**	
	**Total**	**7,21**	**1,777**		**7,76**	**1,832**	
Trust in public health information*	**-49**				**7,69**	**1,930**	**0,007**
	**50–66**				**7,94**	**1,808**	
	**67–79**				**8,16**	**1,607**	
	**80-**				**8,49**	**1,551**	
	**Total**				**7,86**	**1,845**	
Trust in treatment at hospital	**-49**	**7,46**	**1,770**	**< 0,001**	**7,60**	**1,843**	**0,009**
	**50–66**	**7,29**	**1,712**		**7,81**	**1,847**	
	**67–79**	**7,97**	**1,458**		**8,10**	**1,607**	
	**80-**	**8,02**	**1,407**		**8,15**	**1,855**	
	**Total**	**7,50**	**1,713**		**7,76**	**1,814**	
Trust in information at hospital	**-49**	**7,26**	**1,905**	**< 0,001**	**7,30**	**2,028**	**< 0,001**
	**50–66**	**7,07**	**1,875**		**7,55**	**2,162**	
	**67–79**	**7,73**	**1,629**		**7,89**	**1,807**	
	**80-**	**8,03**	**1,552**		**8,68**	**1,117**	
	**Total**	**7,29**	**1,861**		**8,50**	**2,038**	

Bold = significant result (P < .05)

*This item was only used in the 2020 study

(Insert Tables 1 and 2 about here)

To investigate possible changes to trust in healthcare and healthcare actors in the population, we compared findings from the 2019 study with those in the 2020 study (Table 2). There was a statistically significant increased trust in public healthcare, in treatment at hospital, and in information at hospital after the outbreak of the COVID-19 pandemic. There was also a non-significant rise in trust in GPs. The variable with the greatest increase was trust in public healthcare (0.553 on a 1–10 scale, p < .001).

The univariate analysis presented in Table 3 suggests that persons reporting having a chronic disease, had greater trust in their GPs than the population in general. However, when controlling for having received mental health treatment and sociodemographic variables, neither having received mental health care nor having a somatic chronic disease had any significant impact on trust in GP. The only variable that provided a statistically significant explanation to trust in GP was age (Beta = 0.166, p < .001).

The 2020 study demonstrated that neither having received mental health treatment, nor having a somatic chronic disease predicted trust in public health information (Table 4). However, age predicted this.

To further investigate the impact of age on trust, we performed an ANOVA analysis for both the 2019 and the 2020 study (Table 5). The oldest age group reported the highest trust in their GPs, while the young adults (below 50 years) reported the lowest trust. This pattern is similar in the 2019 and the 2020 study. It should be noted that the differences between age groups are significant in all trust scores. However, the significance is weaker in the 2020 study compared to the 2019 study on one of the items, “Trust in treatment at hospital” (2019: p < .001 vs. 2020: p = .009). Hence, the difference between age groups in trust on this matter may have become slightly lower after the outbreak of the COVID-19 pandemic.

## Discussion

The findings confirm that the Norwegian public’s trust in their general practitioner, the public healthcare system, in receiving correct treatment if you become a patient in a hospital, in public health information and in receiving correct information from hospitals, is high. The trust levels are also relatively stable, and even show an increase during the early phases of the pandemic. Norwegians show a greater trust towards in trust in public healthcare services and correct treatment in hospitals after the outbreak of the pandemic (May 2020) than before the COVID-19 outbreak (November 2019) (Table 2). There is also a minor increase in trust towards GPs, but this is not statistically significant. This confirms, in some areas, the ‘rally to the flag-effect.

It is important to note that our study is from a high trust country, and it shows the developments from just before to just after the COVID-19 pandemic, and how the Norwegian society changed between late 2019 and early 2020. Some of the studies mentioned in the background chapter were conducted in low trust countries such as India, USA, Indonesia, Brazil, and Russia [11, 12].

### Trust in general practitioners

Age predicted trust in our study. As demonstrated in Table 5, the older respondents trusted their GPs the most. This was the case both before and during the pandemic. However, it seems that neither having received mental health treatment nor having a chronic somatic disease predicted trust in GPs (Table 3). Other studies have demonstrated lower trust in GPs when patients suffer from mental health issues [33, 35]. In general, anxiety and depression are found to possibly mediate reduced generalised trust [34]. When our study cannot find such a correlation, the conclusion of the previously mentioned studies should be modified.

Trust in GPs did not alter significantly after the outbreak of the pandemic according to our analysis (Table 2). As outlined earlier, trust (or lack thereof) is often a relatively stable entity despite new experiences and societal changes. The 2020 study was made during the very early stage of the COVID-19 pandemic. This unfamiliar and likely stressful situation for chronically ill persons did not reduce their particular trust in their GPs. Patients suffering from chronic diseases are likely to have a greater amount of contact with healthcare professionals. More contact with GPs increases trust levels for this patient group [5], so this may help explain the higher trust they report.

It should be taken into consideration that trust in GPs was very high at baseline (before the pandemic), and that this variable may have reached a ceiling effect. However, the level of trust in GPs is higher than in any other health actors, both before and during the pandemic (Table 2). As demonstrated in other studies, trust in a particular doctor is not necessarily related to the public’s generalised trust in doctors [25–27]. Normally, there is a greater particular trust in particular doctors than generalised trust in doctors as a profession [27, 49]. We would like to emphasise that the question we used to investigate trust in GPs was formulated as “trust your GP”. This item aimed to examine *particular* trust, not generalised trust in doctors.

### Trust in public healthcare and in treatment

We found a statistically significant increase in trust in public healthcare and in treatment in hospitals (Table 2). This rise in public trust in hospitals is perhaps particularly remarkable when viewed against the backdrop of lower activity in Norwegian hospitals during the early phases of the pandemic. Preparing for a surge in COVID-19 patients requiring intensive care, hospitals pre-emptively decreased the activity in other departments and outpatient follow-up. This was well known through the mass media, so the public could be expected to have a *lower* trust in receiving treatment at public hospitals. However, as our study shows, public trust rose instead.

This development of increased trust is in accordance with the findings made by earlier studies that show that trust may increase when society is subject to disasters or emergencies [4]. As described earlier, governmental bodies in high-trust countries have been more successful in controlling the COVID-19 pandemic than low-trust countries [1, 2]. Another possible explanation for the increased trust in Norwegian healthcare institutions, is the strategy of transparency in their public communication [19, 20].

### Trust in health information

Our study shows that the Norwegian public´s trust in public health information increased at the outbreak of the COVID-19 pandemic (Table 2). Trust in public health information was relatively high before, and even higher after the outbreak of the pandemic (Table 2). We found that trust in public health information was not related to mental health nor having a chronic, somatic disease (Table 4). However, Blix et al. (2021) found that being worried about the pandemic is associated with lower generalised trust [50]. Being worried and perceiving the pandemic as a personal threat is related to a general downward adjustment of trust. Harris & Sandal (2021) showed that trust in the healthcare system seemed to act as a protective buffer to worry and psychological distress. At the same time, contracting COVID-19, being medically vulnerable, working in the healthcare system, female gender, younger age, having lower levels of education, or an immigrant background, predicted psychological distress [51].

### Theoretical discussion

We suggest that there is a dynamic relationship between trust in public health information, healthcare institutions, generalised trust and a societal crisis situation, such as the COVID-19 pandemic. Our findings show that the Norwegian public’s trust in health institutions, such as treatment in public hospitals and information from health authorities, rose during the very early stages of the COVID-19 pandemic, while the already very high trust in GPs remained stable. Building on these findings, we will here present a theoretical perspective to highlight how healthcare actors can represent their institutions in a crisis, particularly in the early stages.

Trust is a mechanism for reducing complexity for the individual actors in a society, according to Niklas Luhmann [52]. When health knowledge is lacking, trust in health authorities becomes crucial for cost and benefit assessments related to action choices [53]. If we trust that the authorities know what they are doing, are honest, and have our best interests in mind, we are more likely to follow the advice we are given. High institutional trust can therefore increase compliance with infection control advice.

A crisis such as the COVID-19 pandemic can be assumed to make the population uncertain about the future. However, if the actors representing healthcare institutions behave in certain ways, our findings indicate that trust levels may rise, although this rise is more likely to take place in countries with a relatively high trust in governing institutions. If the authorities normally receive a high degree of (baseline) trust from the population, and in addition communicate in a way that keeps the trust levels stable and high, this may lead to reduced emotional impact of the uncertainty.

Social trust entails a more complex relationship with adherence to recommendations. Research indicate that people are more likely to comply with public health measures when they perceive that others in their social networks are also following these measures [54]. Facing an infectious and potentially lethal virus often elicits natural responses of worry and fear. When individuals feel threatened by an external force beyond their control, fear can arise. Fear towards a threatening stimulus can activate cognitive monitoring systems and influence behavioural and attitudinal change, leading to strategies aimed at minimising risk [55]. Thus, greater uncertainty of the situation may lead to greater trust in the institutions, but a precondition for this is how the representatives of the institutions act and communicate.

Particular trust in GPs seems to be less influenced by events on the societal level. Using the notion of street-level bureaucrats [46], we may describe this trust as a function of the discretionary judgement performed by the doctors in their encounters with the patients. The GP is a representative of what Giddens called “access points of abstract systems” [56], immediately recognisable and far less abstract than the health care system itself. The personal contact between health care personnel and patients is related to greater trust, according to both qualitative and quantitative studies [35, 57, 58]. Further, the communication between patients and GPs has the potential of developing what may be described as more open mandates of trust [48].

Mandates of trust are related to the doctor’s discretionary judgement, and these mandates are the complex result of particular, general and institutional trust levels [48]. The patient’s trust in their GPs is reciprocal, and constantly, but implicitly negotiated [59]. This means the trust in GPs is less likely affected by societal events, such as the COVID-19 pandemic. This is also highlighted in our study. The patients’ trust in GPs is more interpersonal, linked to the patient’s understanding of the doctor’s personal interest, communication skills, and knowledge of modern medicine, plus his or her ability to act on behalf of this knowledge.

The high level government and health officials holding the press conferences during the pandemic, can hardly be described as street level bureaucrats, but they did appear as what Giddens described as access points of healthcare, trying to convey clear and coherent advice [47]. The development of the COVID-19 pandemic and the relevant vaccines, and the rationale for societal restrictions, were explained quite well by Norwegian health authorities. Ihlen et al. indicate that the health care authorities in Norway were largely successful in communicating priorities and policies with the public [20]. They show how health authorities in Scandinavia communicated and built trust during the early stages of the COVID-19 pandemic, and that Norwegian authorities received higher levels of trust than their Swedish and Danish counterparts [20]. The authors argue that this was partly due to differences in what they call transparency management. Transparent communication of uncertainties when giving public advice and presenting research, had a significant positive impact on public trust. Trust appears to decline when stricter measures are being enforced [60]. But in a case study of Norwegian television debates and the public responses they spurred, representatives of the health authorities were more open to participation and better at connecting to everyday experiences of the public than the critics were. The authors concluded that authorities may maintain a high level of trust by “rhetorically cultivating their positions within instrumental and (social) media networks of expertise” [61]. This helped the public form a common perception of the crisis situation, and it may help explain the increased trust in the healthcare institutions. As the authorities displayed their uncertainty, and appeared to be open about the assessments they made, it may have given the regulations legitimacy in great parts of the population [20]. This may be described as discretion, in the way Lipsky uses the term; the decision is not merely based on rules and general principles, but on assessments when facing the users in concrete situations [46].

Our empirical findings highlight the discretionary judgment of representatives of healthcare institutions and the potential impact on the institutions they represent. The particular trust given by the patient to their GPs can be understood as not only a result of the general trust in the GPs as a profession, but also the interpersonal mandates of trust the GPs develop with their patients. This makes the trust in GPs less dependent on the development of the COVID-19 pandemic. The public´s institutional trust in health authorities can be understood as a result of the general trust in healthcare authorities, but also in the more particular trust in the people giving the information, but this is more dependent on generalised trust and risk perceptions during the COVID-19 pandemic.

### Strengths and limitations

The study´s greatest strengths are the snapshots of the Norwegian population’s trust in healthcare just before and just after the outbreak of the COVID-19 pandemic. We planned to do a small study to compare trust in health actors and institutions, but, of course, did not plan for any major crises to occur. By chance, we have captured some aspects of the rally ‘round the flag-effect that would otherwise be very hard to capture without a crystal ball, or very long term samples. Our two studies are based on two separate data collections, covering the same population and using the same statistical bureau, the same questions and samples drawn from the same panel. This gives valuable insights into the changing trust levels just before and just after the outbreak of the pandemic.

A limitation of the study is the nature of surveys; we cannot make casual assumptions based on these data. Furthermore, there is a limitation of the study that it is based on self-reported somatic and mental health. As mentioned earlier, the variable on self-reported mental health is possibly unreliable, as untreated or undiagnosed illnesses may result in inaccurate expressions of the current mental health situation.

It is also a limitation that the samples are not part of a joint research project. This meant that it would be difficult to plan for deductive tests of hypotheses. Thus we had to settle for more abductive reasoning. Further, the 2019 sample and the 2020 sample are slightly different in terms of demographics (Table 1). Still, we find the comparison to be reliable, since these samples are from the same panel, performed by the same company, both have a sample size of > 1000, and data have been weighed to adjust for demographic skewedness.

The indicator for mental health in the 2020 study is not necessarily comparable to previous studies. The question used in the 2020 study is “Have you earlier in your life been treated for mental health issues?” (Yes/No), and it is possible that this does not reflect the persons’ current conditions. The question also poses problems if illnesses are undiscovered, untreated or undiagnosed. 24.6% of the respondents replied “Yes” to this item (Table 2). Thus, this item may be an unreliable indicator for mental health.

## Conclusion

In conclusion, we found that the Norwegian public’s trust in healthcare is high, especially during the early phases of the COVID-19 pandemic. The Norwegian trust in their general practitioner, the public healthcare system, in receiving correct treatment if you become a patient in a hospital, and in receiving correct information from hospitals, remained remarkably high, compared to other countries. We also found that during the COVID-19 pandemic, trust in healthcare institutions increased even further, but this did not necessarily apply for particular trust in GPs. Further, trust could not be predicted by neither somatic nor mental health conditions.

Understanding the factors that influence individual behaviour during a pandemic is critical for effective public health interventions. Institutional, generalised and interpersonal trust all play a role in shaping individual behaviour, and efforts to promote compliance with public health measures should take these factors into account. By continuing to build trust in public health actors and institutions, we can increase the likelihood that individuals will take the necessary steps to protect themselves and others during a pandemic. Health authorities need to know what groups in the population to emphasise during crises. And our study shows that young adults had lower trust in health institutions and their GPs than older adults.

Building the basis for trust in healthcare actors and institutions already begins before a pandemic. Ill-prepared government agencies are not in a good position to be trusted by the public. Perceived risks are important for people’s acceptance of government measures and their adoption of recommended behaviour changes. Our findings show that the personal judgment of healthcare actors can influence the institutions they represent. Trust in a patient’s GP is influenced by trust in the medical profession and the specific relationship between the GP and patient. Similarly, the public’s trust in health authorities is based on trust in the healthcare system and the trustworthiness of the individuals providing information.

More research is needed on the relations between particular, general and institutional trust in healthcare and among healthcare actors.

## Data Availability

The datasets generated and/or analysed during the current study are not publicly available due to the owners considering further publications, but are available from the corresponding author on reasonable request.
